# WALKING AWAY FROM LONELINESS: THE MEDIATING ROLE OF SOCIAL ISOLATION

**DOI:** 10.1093/geroni/igz038.3082

**Published:** 2019-11-08

**Authors:** Natalie Shellito, Nidya Velasco Roldan

**Affiliations:** University of Massachusetts Boston, Boston, Massachusetts, United States

## Abstract

This study examines the relationship between physical activity (PA) and loneliness among older adults. Participation in walking enables individuals to come into contact with other people, thus social isolation may mediate the relationship between walking and loneliness. The study uses participants from the Leave Behind Questionnaire of the 2016 data wave of the Health and Retirement Study with a sample size of 6,157. The dependent variable, loneliness, is measured using the 11-item UCLA Loneliness Scale and the independent variable, walking, is measured as participants who walk 20 minutes or more per day. The mediator, social isolation, is measured using a standardized 9-item score, including closeness and frequency of contact with children, friends, and other family members, and participation in group activities. We analyzed the effect of walking on loneliness and the role of social isolation as a mediator of that relationship using structural equation modeling. Our results suggest that walking is significantly associated with lower levels of both social isolation (B=-.10) and loneliness (B=-.05). As well, there is a positive association between social isolation and loneliness, as social isolation increases, so does loneliness (B=0.31). Moreover, results from the mediation analysis using bootstrapping suggest that social isolation partially mediates the relationship between walking and loneliness (B=-.03). Our findings confirm the benefits of PA on wellbeing. This research provides evidence that suggests establishing walking programs may decrease the risk of loneliness. Future interventions concentrated on lowering social isolation through PA among older adults should consider the opportunity to reduce loneliness.

